# Impact of Tumor Localization and Method of Preoperative Biopsy on Sentinel Lymph Node Mapping After Periareolar Nuclide Injection

**DOI:** 10.1371/journal.pone.0149018

**Published:** 2016-02-11

**Authors:** Julia Krammer, Anja Dutschke, Clemens G. Kaiser, Andreas Schnitzer, Axel Gerhardt, Julia C. Radosa, Joachim Brade, Stefan O. Schoenberg, Klaus Wasser

**Affiliations:** 1 Institute of Clinical Radiology and Nuclear Medicine, University Medical Center Mannheim, Medical Faculty Mannheim, University of Heidelberg, Heidelberg, Germany; 2 Department of Gynaecology and Obstetrics, University Medical Center Mannheim, Medical Faculty Mannheim, University of Heidelberg, Heidelberg, Germany; 3 Department of Gynaecology & Obstetrics, Saarland University Hospital, Homburg, Germany; 4 Institute of Medical Statistics, Biomathematics and Data Processing, Medical Faculty Mannheim, University of Heidelberg, Heidelberg, Germany; INRS, CANADA

## Abstract

**Background:**

To evaluate whether tumor localization and method of preoperative biopsy affect sentinel lymph node (SLN) detection after periareolar nuclide injection in breast cancer patients.

**Methods and Findings:**

767 breast cancer patients were retrospectively included. For lymphscintigraphy periareolar nuclide injection was performed and the SLN was located by gamma camera. Patient and tumor characteristics were correlated to the success rate of SLN mapping. SLN marking failed in 9/61 (14.7%) patients with prior vacuum-assisted biopsy and 80/706 (11.3%) patients with prior core needle biopsy. Individually evaluated, biopsy method (p = 0.4) and tumor localization (p = 0.9) did not significantly affect the SLN detection rate. Patients with a vacuum-assisted biopsy of a tumor in the upper outer quadrant had a higher odds ratio of failing in SLN mapping (OR 3.8, p = 0.09) compared to core needle biopsy in the same localization (OR 0.9, p = 0.5).

**Conclusions:**

Tumor localization and preoperative biopsy method do not significantly impact SLN mapping with periareolar nuclide injection. However, the failure risk tends to rise if vacuum-assisted biopsy of a tumor in the upper outer quadrant is performed.

## Introduction

The occurrence of axillary lymph node metastases in breast cancer patients is an important prognostic factor and has relevant impact on treatment decisions [1–4]. Sentinel lymph node biopsy (SLNB) is well established and accurate for local staging in patients with clinical negative lymph nodes. Before biopsy, lymphoscintigraphy can be successfully used to map sentinel lymph nodes providing detection rates of 85–98% [5–8]. Over the last years continuous changes in the technical performance of sentinel lymph node mapping (SLNM) have been introduced. One of those changes was the shift from a peritumoural towards a periareolar nuclide injection. This injection technique is independent of the palpability of the tumour and therefore feasible for routine application in nuclear medicine. In case the sentinel lymph node (SLN) failed to be recognized and additional intraoperative blue dye injection is unsuccessful surgeons have to consider an axillary dissection for nodal staging.

To prevent the morbidity of extensive axillary surgery it is important to reach a high identification level of SLN. Therefore, physicians have to be aware of patients who are at risk for a failure of SLNM. Multiple studies have evaluated potential risk factors affecting the SLN detection rate using peritumoural injection techniques [9–14], but only little data exist on the use of periareolar nuclide injection. At present, few studies included patients with both, peritumoural and periareolar nuclide injection. The authors identified patient age and tumour size to adversely affect the SLN detection rate. In contrast, tumour localization and prior biopsy were independent factors [15–18].

The purpose of this study was to verify potential risk factors for a decreased SLN detection rate in patients who underwent SLNM with periareolar nuclide injection only. We focused on the influence of tumour localization and method of preoperative biopsy hypothesizing that

1. based on the lymphatic drainage to the axilla the performance of a biopsy in the upper outer quadrant is associated with a higher failure rate of SLN detection compared to other localizations
2. the SLN detection rate is lower in patients with preoperative stereotactic vacuum-assisted biopsy compared to patients who underwent core cut biopsy, as higher tissue damage is caused.

## Patients and Methods

### Breast cancer patients

Our institutional review board (Medizinische Ethikkomission II der Medizinischen Fakultät Mannheim, Heidelberg Universität, Germany) approved this Health Insurance Portability and Accountability Act compliant retrospective study and the need for informed patient consent was waived. All patient information was anonymized and de-identified prior to analysis. 767 patients with biopsy-proven invasive breast cancer were retrospectively included between 01/2008 and 02/2014. Patients with inflammatory cancer or prior extended surgery of the affected breast were excluded from the study. All patients underwent either stereotactic vacuum-assisted biopsy (10G coaxial system) or ultrasound-guided core cut biopsy (14G or 16G coaxial system) for histopathological proof of the tumour. SLNM and following SLNB were performed afterwards at our hospital in all patients. Patient and tumour characteristics were registered as follows: 1) patient age 2) type of biopsy 3) tumour size 4) tumour histology 5) disease focality 6) quadrant-based tumour localization 7) pathological axillary nodal status.

### Lymphoscintigraphy technique and image analysis

In all patients SLNM was performed with periareolar injection of a 99m Tc-labeled colloid. Usually two-day protocols were applied for nuclide injection and surgery (80–100 MBq). In case nuclide injection and surgery occurred on the same day, the amount of injected radio labelled colloid was adapted (40–60 MBq). The sentinel lymph node was located by gamma camera (e.cam VG910b, Siemens Healthcare Sector, Erlangen) approximately 2–3 hours p.i.

SLNs were re-evaluated retrospectively on two plain images by two readers in consensus: one nuclear medicine resident with 4 years experience in breast imaging and one board certified specialist in radiology and nuclear medicine with 15 years experience in breast imaging. The lymphoscintigraphy was categorized depending on the visual signal intensity of the SLN using the following primary categories: either sufficient nuclide uptake with possible skin mark or weak or missing nuclide uptake without a skin mark. The time between biopsy and nuclide injection was registered.

### Statistical analyses

To evaluate the influence of different factors on the sentinel identification rate, Chi-Square-Test and odds ratio (OR) were used. All statistical analyses were performed using SPSS 13.0 statistical package (SPSS 13.0 Inc., Chicago, IL, USA). A p value of <0.05 was considered to be statistically significant.

## Results

767 patients with a median age of 62 years (range 27–90 years) were evaluated. The median tumour size was 17 mm (range 1–100 mm). Multifocal or multicentric disease was found in 134/767 (17.4%) patients. 175/767 (22.8%) patients had axillary lymph node metastases. 61/767 (8.0%) patients underwent vacuum-assisted biopsy and 706/767 (92.0%) patients underwent ultrasound-guided core needle biopsy for histopathological proof of the tumour. The corresponding patient and tumour characteristics are displayed in Table 1 (Data in S1 Data).

**Table 1:** Patients and tumor characteristics (n = 767).

Histology	Number of patients (%)
Invasive ductal, NOS	588 (76.7)
Invasive lobular	90 (11.8)
Mixed	31 (4.0)
Ductal carcinoma in situ	24 (3.1)
Others	34 (4.4)
Tumor localization*	
UOQ	435 (56.7)
UIQ	167 (21.8)
LOQ	114 (14.8)
LIQ	51 (6.7)
**Biopsy type**	
Stereotactic vacuum biopsy	61 (8.0)
Core cut biopsy	706 (92.0)
**Biopsy performed**	
At our institution	416 (54.3)
External	351 (45.7)

* U = Upper; O = Outer; L = Lower; I = Inner; Q = Quadrant

Overall 678 of 767 patients had a sufficient nuclide uptake resulting in a SLN detection rate of 88.4%. In 21 patients additional SLN were found in extra-axillary localizations: 15 internal mammary lymph nodes, 1 pectoral and 1 infraclavicular lymph node. SLN marking failed in 89/767 (11.6%) patients: 49 (6.4%) patients had a weak signal intensity and 40 (5.2%) patients had an absent signal intensity on lymphoscintigraphy.

### Influence of tumour localization and biopsy method

435/767 (56.7%) patients had a tumour in the upper outer quadrant, 332/767 (43.2%) had tumours in other localizations. Sentinel lymph node marking failed in 50/435 (11.5%) patients with a tumour in the upper outer quadrant compared to 39/332 (11.8%) with tumours in other localizations. The failure rate was not significantly correlated with the localization of the tumour (p = 0.9, OR 1.0). Sentinel lymph node marking failed in 9/61 (14.8%) patients with prior vacuum-assisted biopsy and 80/706 (11.3%) patients with prior core needle biopsy. There was no significant correlation between the type of biopsy and the failure rate of SLNM (p = 0.4, OR 1.4).

SLNM failed in 7/32 (21.9%) patients who underwent vacuum-assisted biopsy of a tumour in the upper outer quadrant compared to 2/29 (6.9%) patients who underwent vacuum-assisted biopsy of a tumour in other localizations. The corresponding OR of failure of SLNM showed a trend with 3.8, but Chi-Square test did not reach statistical significance in this subset of patients (p = 0.09). If core needle biopsy was performed SLNM failed in 43/403 (10.7%) patients who had a tumour in the upper outer quadrant compared to 37/303 (12.2%) patients with a tumour in other localizations. There was no significant correlation between the tumour localization in the upper outer quadrant and failure rate of SLNM (p = 0.5, OR = 0.9) in this biopsy group. The tumour localization in each biopsy group and the corresponding SLN detection rates are itemized in Table 2 (Data in S1 Data).

**Table 2:** Influence of tumor localization and biopsy method on the SLN detection rate (n = 767).

Factor		SLN identified (%)	SLN not identified (%)	Significance	Odds ratio (95% confidence interval, CI)
Tumor	Tumor UOQ	385 (88.5)	50 (11.5)	p = 0.91	1.0 (CI 0.6–1.5)
localization	Other quadrant	293 (88.2)	39 (11.8)		
Biopsy type	Vacuum biopsy	52 (85.2)	9 (14.8)	p = 0.42	1.4 (CI 0.6–2.8)
	Core biopsy	626 (88.7)	80 (11.3)		
Vacuum	Tumor UOQ	25 (78.1)	7 (21.9)	p = 0.09	3.8 (CI 0.7–19.9)
biopsy and	Tumor in other quadrant	27 (93.1)	2 (6.9)		
Core biopsy	Tumor UOQ	360 (89.3)	43 (10.7)	p = 0.52	0.9 (CI 0.5–1.4)
and	Tumor in other quadrant	266 (87.8)	37 (12.2)		

### Other potential influence factors

To evaluate the influence of time between biopsy and nuclide injection, the median time interval of 14 days (range 2–236 days) was set as threshold in our study population. Performing the nuclide injection in less than 14 days compared to 14 days or more after biopsy did not significantly affect the success rate of SLNM in patients with prior vacuum-assisted biopsy (OR = 1.9, p = 0.4) or core needle biopsy (OR = 1.1, p = 0.8).

To assess the influence of patient age, 50 years was set as threshold limit. This age represents the shift in menopausal status, which is accompanied by an increasing breast involution. SLNM failed in 81/629 (12.9%) patients aged 50 years or more compared to 8/138 (5.8%) patients younger than 50 years. A patient age of ≥ 50 years was associated with a significant decrease in the SLN detection rate (p = 0.02, OR = 2.4). Additional evaluated parameters, which did not reach statistical significance for the failure rate of SLNM, were tumour size (p = 0.14), disease focality (p = 0.65) and histological lymph node status (p = 0.21). The described potential risk factors and the corresponding SLN detection rates are itemized in Table 3 (Data in S1 Data).

**Table 3:** Additional potential factors influencing the SLN detection rate (n = 767).

Factor		SLN identified (%)	SLN not identified (%)	Significance	Odds ratio(95% confidence interval, CI)
Time between vacuum biopsy and nuclide injection	<14 days	11 (78.6)	3 (21.4)	p = 0.42	1.9 (CI 0.4–8.7)
	≥14 days	41 (87.2)	6 (12.8)		
Time between core biopsy and nuclide injection	<14 days	257 (88.3)	34 (11.7)	p = 0.80	1.06 (CI 0.7–1.7)
	≥14 days	369 (88.9)	46 (11.1)		
Age	≥50 years	548 (87.1)	81 (12.9)	p = 0.02	2.4 (CI 1.1–5.1)
	<50 years	130 (94.2)	8 (5.8)		
Size	>T2	24 (80.0)	6 (20.0)	p = 0.14	1.9 (CI 0.8–5.0)
	≤T2	654 (88.7)	83 (11.3)		
Focality	Unifocal	558 (88.2)	75 (11.8)	p = 0.65	1.2 (CI 0.6–2.1)
	Multifocal/ multicentric	120 (89.6)	14 (10.4)		
Axillary nodal status	Positive	150 (85.7)	25 (14.3)	p = 0.21	1.4 (CI 0.8–2.3)
	Negative	528 (89.2)	64 (10.8)		

## Discussion

To ensure a sufficient axillary staging with SLNB in breast cancer patients the knowledge of potential risk factors that could decrease the detection rate of the SLN is of high clinical importance.

To our knowledge this is the first study assessing the impact of prior vacuum-assisted biopsy versus core needle biopsy as well as tumour localization on SLNM after periareolar nuclide injection. We could demonstrate that the interaction of a prior vacuum-assisted biopsy and a tumour localization in the upper outer quadrant adversely affected SLNM success with a 3.8 time higher risk of failure (OR = 3.8, p = 0.09) compared to tumours in other localizations. This was not the case if core needle biopsy was performed. Despite a higher risk of failure statistical significance was not reached in patients with prior vacuum-assisted biopsy. A reason for this could be the overall low number of included patients in this subgroup (n = 61). Further, the odds ratio of 3.8 is accompanied by a relatively wide confidence interval and therefore more likely represents a tendency. Studies have described that patients undergoing excisional biopsy for breast cancer can have modifications of the drainage pathway from the breast towards the axilla [19]. It is possible, that these changes occur after performing a vacuum-assisted biopsy as well. Compared to core biopsy, larger needle diameters, more frequent tissue sampling as well as the vacuum effect may cause extended trauma to the breast. It is further known that lymphatics in patients with untreated breast cancers predominantly drain towards the axillary nodes [20] and a tumour localization in the upper outer quadrant might additionally interfere with an unimpeded lymph drainage. We assume, that the combination of these conditions might negatively influence the lymph flow and the nuclide transport from the breast towards the axillary nodes leading to a decreased SLN detection rate. In the study of Chagpar et al. neither prior needle biopsy nor excisional biopsy had a negative impact on the detection rate of the SLN [15], but information on the type of needle biopsy was not given. Gschwantler-Kaulich et al. confirmed that prior core cut biopsy did not decrease the SLN detection rate and that the tumour localization itself was an independent factor in both studies [16]. However, comparison is limited due to the fact that varying injection techniques were performed in their study populations.

At present, only little data exists on the impact of timing between biopsy and following nuclide injection and recommendations from the guidelines still have to be defined. Haigh et al. described no significant impact of the time interval from biopsy in patients with peritumoural nuclide injection, but they did not include patients with prior vacuum-assisted biopsy in their study [21]. Using periareolar nuclide injection we demonstrated that the time interval between biopsy and nuclide injection had no statistical significant influence on the SLN detection rate, regardless of whether prior vacuum-assisted biopsy (p = 0.4) or core needle biopsy (p = 0.8) was performed. With an OR of 1.9, we just found a slightly increased risk of a failure in SLN detection, if lymphoscintigraphy was performed within the first 14 days after vacuum-assisted biopsy. It can be assumed that given the pronounced acute post biopsy changes a prior vacuum-assisted biopsy can potentially increase the failure rate of detection, if a SLNM is performed too close from breast biopsy.

Regarding patient age, we found a significantly higher rate of insufficient SLNM (12.9% vs. 5.8%, p = 0.02) in patients aged ≥50 years. This confirms results of other studies. Gschwantler-Kaulich et al. [16] provided a year-by-year analysis and found a 4% decrease of OR to fail per additional year. Chagpar et al. [18] and Motomura et al. [17] described a higher failure rate in patients who were 60 years of age or older compared to younger patients (4.7% vs. 7.3% and 1.6% vs. 5.6%). In their study the authors do not explain, why the age of 60 was set as threshold in their study. To our opinion it is more useful to define the threshold at the age of 50, which more likely represents hormonal changes, changes of the breast tissue and maybe even changes of the lymph node structure. Following, the higher rate of insufficient SLNM might be due to a proceeding fatty alteration of lymphatic tissue at higher ages, which could decrease the lymph nodes’ ability to accumulate the radiocolloid.

There is controversial literature on the impact of the tumour size on SLNM. Some authors report a positive correlation between the detection rate and tumour size [16, 18, 22], others describe a negative correlation [23]. In our study the tumour size had no influence on the SLN detection rate using periareolar nuclide injection. This is in line with other studies using periareolar and peritumoural injection techniques [9, 17, 21, 24].

Further, the nodal status and disease focality were no relevant risk factors, which confirms the findings of Gschwantler-Kaulich et al. [16] and Schrenk et al. [25] including periareolar injection techniques.

Our study had several limitations: First, our analysis was performed retrospectively at a single institution and we cannot draw definite conclusions based on our data. Following, there is a need for prospective studies with representative patient populations to confirm our results. Second, the sample size of patients with vacuum-assisted biopsy was small, which lowers statistical power. This is caused by the fact, that solid lesions were usually biopsied under ultrasound guidance. In case a correlate on ultrasound was missing, but microcalcifications were registered on mammograms patients underwent vacuum-assisted biopsy. Histopathology frequently showed ductal carcinomas in situ in these patients and SLNM was usually not performed. Following the sample size of patients with invasive breast cancer and vacuum-assisted biopsy was diminished. Further, as the patient age was a significant factor for a decreased SLN detection rate the addition of a multivariate analysis would have been desirable in these patients, but could not be performed due to the small number of patients with failed SLNM aged younger than 50 years. Last, a final correlation of the SLN detection rates on lymphoscintigraphy with the intraoperative detection rates was not available in our study setting.

## Conclusion

We can conclude, that the tumour localization and method of preoperative biopsy do not significantly affect the sentinel lymph node detection rate. However, performing a vacuum-assisted biopsy of a tumour in the upper outer quadrant may be a potential risk factor for a failure of SLNM after periareolar nuclide injection. Our findings should not indicate to avoid a routine SLNM with periareolar nuclide injection if the described conditions were met. But physicians should pay extended attention and consider adaptions in the clinical workflow, such as an additional peritumoural nuclide application, in time. Future studies should verify these results in larger patient populations.

## Supporting Information

S1 Data Data set of study population. (XLS)
